# Genomic Insights Into Population Structure and Demographic History of White Anglerfish ( *Lophius piscatorius* ) Throughout Its Range

**DOI:** 10.1111/eva.70288

**Published:** 2026-07-25

**Authors:** Atal Saha, María Quintela, Kjell Nedreaas, Naiara Rodríguez‐Ezpeleta, François Besnier, Hans Gerritsen, Halvor Knutsen, Rita Castilho, Karen Agatha Martínez‐Swatson, Kevin Glover, Per Erik Jorde

**Affiliations:** ^1^ Department of Natural Sciences, Centre for Coastal Research University of Agder Kristiansand Norway; ^2^ Institute of Marine Research Bergen Norway; ^3^ AZTI, Marine Research Basque Research and Technology Alliance (BRTA) Sukarrieta Bizkaia Spain; ^4^ Fisheries Ecosystems Advisory Services Marine Institute Galway Ireland; ^5^ Institute of Marine Research, Flødevigen Research Station Arendal Norway; ^6^ Universidade do Algarve, Campus de Gambelas Faro Portugal; ^7^ CCMAR/CIMAR ‐ Centro de Ciências do Mar do Algarve, Campus de Gambelas Faro Portugal; ^8^ Pattern Institute Faro Portugal; ^9^ GRID‐Arendal Tyholmen Norway

**Keywords:** conservation genetics, marine genomics, monkfish, sustainable management

## Abstract

Understanding population structure and clarifying species boundaries are essential for the sustainable management of exploited marine species. Although genomic resources for shallow‐water taxa have expanded rapidly, open‐water species remain comparatively understudied. Here, we present a comprehensive genomic analysis of the white anglerfish ( *Lophius piscatorius* ), a commercially important species that occupies a wide depth range. We examined genetic structuring and verified species identity across its distribution range in the North Atlantic and Mediterranean. We combined whole‐genome sequencing of selected individuals with targeted SNP genotyping of 897 specimens from 35 locations. Our results support a panmictic or nearly panmictic population of white anglerfish throughout the Northeast Atlantic, consistent with earlier studies, here extended northward to about 68° N. We found no evidence of local adaptation or selection. Like earlier studies, we detected apparently misclassified specimens of the closely related black anglerfish ( *L. budegassa* ) and extensive hybridization with up to 20% hybrids in local samples from around the Celtic Sea and nearby waters. Most or all hybrids appear to be first generation (F1), with a few potential backcrossed individuals of less certain identification. Demographic analyses indicate a recent colonization of the North Atlantic, with black anglerfish diverging more recently and maintaining a smaller effective population size. These results underscore the value of integrating genomic tools into monitoring frameworks to improve species identification, delineate population units, and inform sustainable fisheries management.

## 1 Introduction

Ensuring the long‐term sustainability of fisheries requires the persistence of viable wild populations, yet this goal is increasingly challenged by anthropogenic pressure such as overfishing, climate change, and pollution. A reliable management is one of the requisites of sustainability and needs to be based, among other pillars, on an accurate definition of units/stocks (Ryman et al. 1995; Kerr et al. 2017). The idea that marine populations exhibit low genetic differentiation due to high dispersal capabilities and the lack of physical barriers in the ocean has been repeatedly challenged (e.g., Hauser and Carvalho 2008; Salmenkova 2011). Traditionally, fisheries stocks have often been delineated upon political and administrative considerations (Stephenson 2023), often leading to misalignment with the biological units (Reiss et al. 2009; Kerr et al. 2017; Quintela et al. 2020). Over recent decades, the growing application of genetic and, more recently, genomic markers for stock definition (Casey et al. 2016 and references therein) has uncovered substantial genetic structuring in many shallow‐water marine species. In contrast, open‐ and deep‐water species remain relatively understudied, leaving important gaps in our understanding of their genetic diversity, evolutionary dynamics, population structure and implications for management.

Some marine species, particularly those inhabiting deep or open waters, tend to exhibit limited genetic structure across broad geographic ranges (Lundy et al. 1999; Ferrari et al. 2023; Quintela et al. 2024). This pattern is typically explained by high dispersal potential during early‐life stages, the absence of strong physical barriers in the marine environment, and large effective population sizes that minimize the influence of genetic drift (Palumbi 1994; Ward et al. 1994). Additionally, species in the Northeast Atlantic recolonized their current habitats relatively recently, following the Last Glacial Maximum, leaving little time for substantial postglacial population divergence to occur (Hewitt 2000). The combination of short evolutionary time and large population size typical of marine species hinders the detection of population structure, namely with neutral genetic markers alone. This fueled the use of non‐neutral markers to delineate population units within marine fishes (André et al. 2011; Jorde et al. 2018; Breistein et al. 2022), as well as to evaluate the biological validity of existing management units and guide effective conservation and fisheries planning.

White anglerfish ( *Lophius piscatorius* Linnaeus, 1758) is found in a wide range of water depths throughout the Northeast Atlantic and Mediterranean Sea. Spawning seasonality varies according to geographic areas (Fariña et al. 2008), with females maturing at a larger size than males. The fertilization is external, with a common reproductive strategy for the genus, that is, the release of buoyant gelatinous egg masses in single veils, which not only facilitate dispersion but also protects against predators. *L. piscatorius* produces a single batch during the spawning season (Afonso‐Dias and Hislop 1996) and its dispersal capacity is foreseen to be large owing to an extended pelagic phase during which eggs and larvae can drift up to 4 months before settling on the seabed (Russell 1976; Hislop et al. 2001). In addition, juveniles and adults are known to move considerable distances, although the purpose of such movements is not fully understood. In the NE Atlantic, mark‐recapture experiments showed displacements of 292 km, from Santander in the Cantabrian Sea (Spain) to the coast of La Rochelle (France) (Landa and Pereda 1997), whereas Laurenson et al. (2005) reported displacements of up to 876 km by a tagged immature female, from Shetland Islands to SE Iceland, showing that large movements are not exclusive of mature individuals. The species' high mobility was also confirmed by Laurenson (ICES 2018) who reported an anglerfish measuring 104 cm, caught off the Norwegian coast in 2006, which had been tagged in 2000 nearly 500 km away, west of Shetland at a length of 45 cm, and had remained at liberty for 5 years and 9 months. This observation suggests mixing of stocks while informing on the growth rate of the species. Both the drifting phase and the movements of juveniles and adults are expected to facilitated gene flow and thus leave an imprint on patterns and magnitude of genetic structure. The bathymetric distribution of the species is from shallow inshore waters (≈20 m depth) down to depths of 1000 m (Caruso 1986), in a variety of habitats such as sandy or muddy substrate and even in kelp. Vertical displacements of immature and mature fish from the bottom to near the surface have been documented in the NE Atlantic, and whereas their cause is unknown, they have been speculated to relate to either feeding or spawning (Hislop et al. 2000).

The literature on genetics of *L. piscatorius* starts back in the 1980s when 11 allozyme loci revealed differentiation between Scotland and the Irish Sea as well as low genetic variation off the west coast of Scotland (Crozier 1987). Nonetheless, a following study based on nine microsatellites revealed no evidence of spatial or temporal differentiation among samples from Iceland, Norwegian coast, the Northern Shelf, Rockall, and West of Ireland (O'Sullivan et al. 2006). Microsatellite loci also reported high levels of polymorphism not only for *L. piscatorius* but also for the partially sympatric black anglerfish ( *L. budegassa* Spinola, 1807) from the Cantabrian Sea, north of Spain (Blanco et al. 2008). Mitochondrial control region sequenced in both white and black anglerfish revealed limited genetic structure and some evidence of a demographic expansion that probably happened early in the Pleistocene before the Last Glacial Maximum (Charrier et al. 2006). The apparent lack of isolation by distance in the two species seemed to be driven by high larval dispersal capacities. A recent RAD‐seq based study by Aguirre‐Sarabia et al. (2021) suggested that white anglerfish in the Northeast Atlantic constitute a single panmictic population.

White and black anglerfish show a broad geographic overlap in the Northeast Atlantic, particularly from the Iberian Peninsula through the Bay of Biscay and into parts of the Celtic Seas, where both species co‐occur on continental shelf and slope habitats. Their spawning areas also overlap substantially in these regions, with both species reproducing along the shelf edge and upper slope, although black anglerfish tend to have a more southerly distribution and spawning peak compared to white anglerfish (Afonso‐Dias and Hislop 1996; Fariña et al. 2008). This spatial and temporal overlap in spawning grounds provides opportunities for interspecific encounters and hybridization. These two species naturally hybridize, leading to up to 20% hybrids in certain areas (Aguirre‐Sarabia et al. 2021). Due to their highly similar external morphology, they are often misidentified in the fishery. Species mislabeling in the market has been documented using genetic approaches, notably through both SNP analyses (Aguirre‐Sarabia et al. 2021) and polymerase chain reaction–restriction fragment length polymorphism techniques (PCR‐RFLP; Armani et al. 2012). These close morphological and genetic similarities may reflect a relatively recent divergence from a common ancestor—an aspect that could be more fully elucidated through comprehensive whole‐genome analyses.

The white anglerfish provides an example of mismatch between stock delineation and putative biological units. The species is currently assessed in the Northeast Atlantic as six separate stocks: the Northeast Arctic stock in ICES subareas 1 and 2 (mostly 2a; Nedreaas 2020), the Northern Shelf stock (Skagerrak, Kattegat, North Sea, West of Scotland and Rockall), the Central Celtic Seas and Northern Bay of Biscay Stock, and the Southern Stock (Atlantic and Iberian waters) in addition to national stocks at Iceland and the Faroes (Thangstad et al. 2006; ICES 2022, 2024a, 2024b). This occurs despite the lack of support for such a stock structure provided by molecular markers (Aguirre‐Sarabia et al. 2021; Blanco et al. 2008; Charrier et al. 2006; Crozier 1987; O'Sullivan et al. 2006), mark‐recapture experiments (Landa, Duarte, and Quincoces 2008; Laurenson et al. 2005), and otolith shape analysis (Cañás et al. 2012). The situation is further complicated by the fact that management does not take place at the species level: Total Allowable Catches (TACs) are set for the white and black anglerfish combined because the landings are often not reported by species. Additionally, the management areas do not match the assessment areas. These mismatches make sustainable management particularly challenging given the species' high commercial value and heavy exploitation by trawl and gillnet fleets from Portugal, Spain, France, Ireland, UK, Denmark, and Norway.

This study aimed to improve understanding of population structure and connectivity in white anglerfish across its Northeast Atlantic distribution and to help identify biologically meaningful stock units for fisheries management. We tested for genetic differentiation consistent with spatial population structuring across the species' range. Whole‐genome sequencing was used to characterize genome‐wide variation and develop a reduced SNP panel for geographically broader sampling. These data were integrated with published RAD‐seq data (Aguirre‐Sarabia et al. 2021), providing coverage across most of the species' distribution, from the Mediterranean Sea to northern Norway.

## 2 Material and Methods

### 2.1 Sampling

**FIGURE 1:**
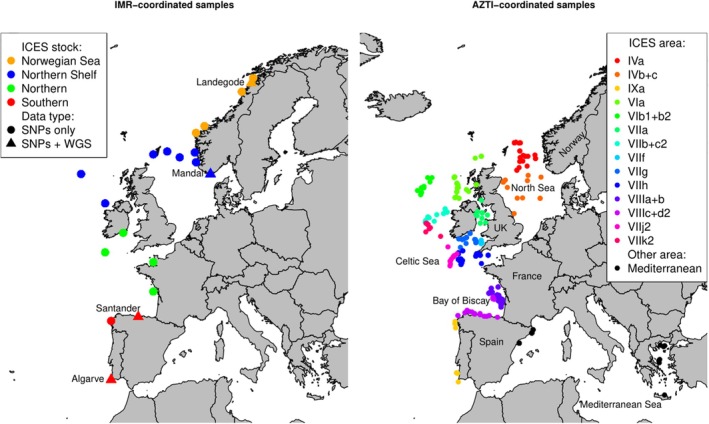
Sampling locations of the present investigation. Left panel contains samples that were collected by IMR for this study (average positions for sampled individuals): dots identify samples that were used for SNP genotyping whereas triangles identify samples that were subject to whole‐genome sequencing (WGS) with colors following ICES stock membership. Right panel: Dots refer to positions of individual fishes from the AZTI‐coordinated samples, previously included in the Aguirre‐Sarabia et al. (2021) analyses, color‐coded according to ICES statistical areas. All individuals from both IMR and AZTI collections were screened for 231 SNPs. See Table 1 for sample details. Figure drawn using *maps* R‐package (Becker et al. 2025).

Morphologically identified white anglerfish were obtained from various sources, which included the reuse of samples collected for other purposes. Briefly, a set of 564 individuals from 20 localities (left panel in Figure 1) was assembled by the Institute of Marine Research in Norway (IMR) with a focus on the northern region, including 142 individuals collected from Norwegian waters during 2016–2019, originally sampled for assessment of chemical contaminants (Frantzen et al. 2020) and stored freeze‐dried. Commercial vessels from the Norwegian coastal reference fleet supplied an additional 123 individuals collected in 2021 and stored as fin‐clips on ethanol. Finally, 299 individuals from outside Norwegian waters were obtained through a collaborative effort of research institutions based in France, Ireland, Norway, Scotland, and Spain via devoted scientific cruises, and from fish markets in A Coruña (Spain) and Algarve (Portugal) as catches from the coastal artisanal fleet during 2021 and stored as muscle tissue in ethanol. The set of 564 IMR individuals was completed with 333 individuals (hereafter, the AZTI‐coordinated samples, which also included known black anglerfish) previously analyzed by Aguirre‐Sarabia et al. (2021) (right panel in Figure 1) and described therein. All samples are summarized in Table 1.

**TABLE 1:** Sample collections of anglerfish (*Lophius* sp., with mean geographic coordinates for each sample. Sample sizes (*n*), unbiased estimated heterozygosity (*H*_S_), and inbreeding coefficients (*F*_IS_) averaged over 231 SNPs (Nei and Roychoudhury 1974) are shown separately for the whole material (*n* = 897) and for *L. piscatorius* alone (*n* = 772) after eliminating inferred *L. budegassa* and hybrids. AZTI‐coordinated samples (here grouped according to ICES statistical areas) are from Aguirre‐Sarabia et al. (2021) and have been collected by a number of fisheries labs; IMR‐coordinated samples are previously unpublished. *) Samples also included in the whole‐genome dataset.

Sample	Position		*Lophius* sp.			*L. piscatorius* only		
	Lat	Long	*n*	*H*_S_	*F*_IS_	*n*	*H*_S_	*F*_IS_
IMR samples								
Steigen	67.8	14.6	25	0.332	−0.000	25	0.332	−0.000
Landegode*	67.3	14.3	34	0.333	0.005	34	0.333	0.005
Lurøy	66.4	12.7	30	0.328	0.008	30	0.328	0.008
Elnesvågen	62.9	6.5	25	0.328	0.007	25	0.328	0.007
Stadt	62.2	5.1	25	0.321	0.056	24	0.326	0.051
North Sea west	60.3	0.3	25	0.334	0.015	25	0.334	0.015
Sotra	60.2	4.9	25	0.329	0.041	25	0.329	0.041
Scotland	60	−2	26	0.327	0.040	24	0.331	0.040
North Sea east	59.7	2.4	17	0.311	−0.031	17	0.311	−0.031
Skudenes	59.2	5.1	25	0.326	0.013	25	0.326	0.013
Mandal*	58	7.5	34	0.328	0.069	34	0.328	0.069
Rockall	58	−14	29	0.318	0.018	29	0.318	0.018
Ireland north coast	55	−10	30	0.321	−0.001	29	0.325	−0.005
Celtic Sea north	52	−7	30	0.312	0.054	26	0.323	0.017
Celtic Sea central	50	−10	30	0.292	0.061	26	0.318	0.019
Roscoff	49	−2	30	0.298	0.066	25	0.322	0.039
Gulf of Biscay	46	−2	31	0.320	0.046	30	0.328	0.035
Santander*	43.4	−4.5	31	0.324	−0.012	31	0.324	−0.012
Malpica	43	−9	30	0.327	0.008	28	0.335	−0.006
Algarve*	37	−9	32	0.064	0.151	3	0.307	−0.042
AZTI samples								
ICES IVa	60.1	1.4	28	0.328	−0.019	28	0.328	−0.019
ICES IVb + c	56.5	1.0	14	0.324	0.019	14	0.324	−0.019
ICES VIa	57.0	−8.4	32	0.320	−0.033	30	0.327	−0.037
ICES VIb1 + b2	56.7	−14.7	10	0.326	−0.062	10	0.326	−0.062
ICES VIIa	53.8	−5.2	12	0.341	0.006	12	0.341	0.006
ICES VIIb + c2	53.6	−13.1	19	0.303	0.012	17	0.322	−0.008
ICES VIIf	51.1	−5.3	10	0.326	−0.017	10	0.326	−0.017
ICES VIIg	51.4	−7.4	23	0.315	−0.022	20	0.332	−0.028
ICES VIIh	49.5	−7.2	47	0.287	−0.020	34	0.321	−0.041
ICES VIIIa + b	45.4	−2.6	63	0.225	0.093	32	0.333	0.002
ICES VIIIc + d2	44.0	−5.5	20	0.305	−0.020	16	0.334	−0.037
ICES VIIj2	49.4	−10.1	13	0.312	−0.019	11	0.328	−0.024
ICES VIIk2	52.7	−14.0	14	0.291	−0.031	10	0.324	−0.048
ICES IXa	39.7	−9.3	7	0.260	0.155	5	0.324	0.049
Mediterranean	40.7	9.9	21	0.162	0.246	8	0.297	0.009
Pooled			897	0.303	0.036	772	0.328	0.005

### 2.2 DNA Extraction, Library Preparation, and Whole‐Genome Sequencing

For the IMR‐coordinated samples (Table 1), DNA was extracted using the Qiagen DNeasy 96 Blood & Tissue Kit in 96‐well plates on a Hamilton Microlab STAR robotic workstation, each containing two or more negative controls, following the manufacturer's protocol. DNA samples were then normalized to homogenize concentration before proceeding. Whole‐genome sequencing was performed on a total of 55 individuals, taken from four sample localities covering the north–south sample range: Algarve (*n* = 14) and Santander (14) on the Iberian Peninsula; and Mandal (13) and Landegode (14) in Norway. Illumina DNA sample preparation and 2 × 150 bp paired‐end sequencing was done commercially on a NovaSeq S4 platform at the Norwegian High Throughput Sequencing Centre, Oslo, Norway.

### 2.3 Whole‐Genome Sequencing

Sequencing reads from the 55 individuals were aligned to the white anglerfish reference chromosome‐level assembly (Dubin, A., Nord University, Bodø, Norway, personal communication) using BWA mem v2 (Li and Durbin 2009). Alignment was followed by sorting using SAMtools v1.8 (Li et al. 2009). No data filtering was applied prior to mapping against the reference genome. The alignment process produced two BAM files per individual (one per lane), which were merged using PICARD's v2.27.1's MarkDuplicates tool (https://broadinstitute.github.io/picard/). PCR duplicates were flagged using the same tool. The quality of the resulting BAM files was evaluated with Qualimap v2.2.1 (García‐Alcalde et al. 2012).

Individual genomic variant call format (gVCF) files were generated using the HaplotypeCaller from the Genome Analysis Toolkit (GATK) v4.2.6.1 (McKenna et al. 2010). Joint genotyping of all samples was performed with GATK's GenotypeGVCFs. To filter low‐quality variants, we applied a hard filter approach using GATK's VariantFiltration tool. SNPs were filtered based on the following thresholds: QD < 2.0, MQ < 40.0, FS > 10.0, MQRankSum < −5.0, ReadPosRankSum < −5.0, and SOR > 5.0. For indels, the criteria were: QD < 2.0, FS > 10.0, ReadPosRankSum < −5.0, and SOR > 5.0. VCFtools v0.1.16 (Danecek et al. 2011) was used to retain only biallelic SNPs with a minor allele count (MAC) ≥ 2. Additionally, variants from unassigned scaffolds were removed. We annotated the ID field of the joint VCF file using BCFtools v1.8 (Danecek et al. 2021). A maximum of 30% data incompleteness was allowed per SNP, and the mean read depth ranged from 5 to 100. Loci that grossly deviated from Hardy–Weinberg equilibrium (HWE, *p* ≤ 0.0001) were excluded. Finally, loci in linkage disequilibrium (LD > 0.5) were removed, leaving nearly 3.8 million (3,777,012) SNPs for the whole‐genome downstream analyses.

### 2.4 Design of SNP Panel and Genotyping

Using the whole‐genome sequences, we designed a smaller SNP panel for genotyping the remaining IMR‐coordinated samples and localities (cf. Table 1). For this panel we selected SNPs with MAF > 0.1, mean depth of coverage > 10, and without polymorphic sites within 70 bases flanking regions. This yielded 243 SNPs that were then turned into nine assays of 27 SNPs for screening the remaining individuals on a Fluidigm platform (Fluidigm Corporation, South San Francisco, CA, USA). Of the 243 SNPs screened in the total material, 231 turned out to be polymorphic in our sample collections, and downstream analyses used these 231 SNPs which cover all 23 chromosomes (Figure S1).

### 2.5 Harvesting ddRAD Sequencing Data

Archived ddRAD sequencing data from Aguirre‐Sarabia et al. (2021) were utilized for SNP detection in anglerfish. Sequence reads from 333 individuals were processed in STACKS v2.62 (Catchen et al. 2013) using the *ref_genome* function. Reads were quality‐checked and trimmed with the *process_rad‐tags* module. Processed reads were mapped to the white anglerfish reference assembly, as we did for the whole‐genome sequences (above), using BWA mem v2 (Vasimuddin et al. 2019). The *gstacks* module was used to build loci from the aligned paired‐end data, and the populations module exported VCF files. VCFtools v0.1.16 (Danecek et al. 2011) was applied to retain only biallelic SNPs with a MAC ≥ 2. Data were further filtered using the same criteria as the whole‐genome sequencing data.

### 2.6 Analyzing Population Structure

We conducted principal component analysis (PCA) using the R (R Core Team 2025) package adegenet v2.1.1 (Jombart 2008) on the SNP data (comprising 231 SNPs genotyped across 897 individuals) and separately on the whole‐genome dataset (3,777,012 SNPs, 55 individuals). This step confirmed the observation by Aguirre‐Sarabia et al. (2021) that several of the sampled individuals were of the closely related *L. budegassa* (black anglerfish) as well as potential hybrids. Individuals were then assigned to species based on 231 SNP genotypes with the STRUCTURE software (Pritchard et al. 2000), running 1 million MCMC repeats after a burn‐in period of 100,000, assuming *K* = 2 populations in an ADMIX model with correlated allele frequencies. Individuals with a high *q*‐value (*q* > 0.93) were assigned to the respective species, whereas individuals with intermediate *q*‐values were tentatively considered as hybrids. Once removed the individuals regarded as black anglerfish as well as putative hybrids, a total of 772 white anglerfish individuals remained. Spatial genetic structure was assessed for the 772 white anglerfish using the SNP data. For spatial analyses, AZTI samples were grouped into ICES statistical areas, pooling neighboring areas with small samples as in Table 1 (i.e., ICES area IVb with IVc, VIb1 with VIb2, VIIb with VIIc2, VIIIa with VIIIb, and VIIIc with VIIId2). Along with the 20 IMR sampling localities, this resulted in a total of 35 localities for spatial analyses. Geographic coordinates were assigned by averaging individual coordinates within each locality where available (Table 1). Unbiased estimates of expected heterozygosity (*H*_S_) and fixation index (*F*_IS_) were estimated within each sample as well as for the total material using expressions provided by Nei and Roychoudhury (1974).

Genetic differentiation among samples was evaluated by pairwise *F*_ST_ (Weir and Cockerham 1984), calculated with VCFtools on the whole‐genome data and with Genepop v4.8.2 (Raymond and Rousset 1995; Rousset 2008) on the SNP panel data. Tests for genetic differences were carried out on 231 SNP allele frequencies using 2 × 2 contingency chi‐square tests among pairs of samples, omitting any SNP with expected value < 1. Test results (*X* ^2^) were summed over SNPs for a joint test of the null‐hypothesis of no differentiation and evaluated against the theoretical chi‐square distribution with df = number of SNPs (Ryman and Jorde 2001). Test results were evaluated separately and by the false discovery rate (FDR) approach, following Benjamini and Yekutieli (2001) as implemented in the *p.adjust* function in R. Finally, we pooled samples belonging to different ICES management stocks (leaving out the Mediterranean samples) and calculated pairwise *F*_ST_ and tested for allele frequency differences among units as described above.

To search for potential larger scale population structure, we investigated if North Atlantic samples adhered to a pattern of isolation by distance (IBD) by confronting pairwise *F*_ST_ against shortest water distance measured with the *marmap* package (Pante and Simon‐Bouhet 2013) in R. Trends were tested for significance with the mantel.rtest in the ade4 package (Dray and Dufour 2007).

The relationship among four of the ICES stocks in which the species is assessed was examined using the discriminant analysis of principal components (DAPC; Jombart et al. 2010) implemented in the R (R Core Team 2025) package *adegenet* v.2.1.11 (Jombart 2008). To avoid overfitting, both the optimal number of principal components and discriminant functions to be retained were determined using the cross‐validation function (Jombart and Collins 2015; Miller et al. 2020).

### 2.7 Scan for Selection in White Anglerfish

Manhattan plots of pairwise *F*_ST_ values were generated for the whole‐genome data using the R package qqman v0.1.4 (Turner 2014). SNPs with *F*_ST_ values exceeding the 99.95th percentile were initially classified as outliers (e.g., Saha et al. 2022). To justify the selection context of these outliers, we used the “intersect” function in bedtools v2.27.1 (Quinlan and Hall 2010) to determine whether any were located within functional regions of the genome.

Outlier detection approaches implemented in BayeScan (Foll and Gaggiotti 2008) and Arlequin (Excoffier et al. 2007) were used to identify potential outlier loci, with default settings for both programs, for the dataset consisting of 772 individuals genotyped at 231 SNPs.

### 2.8 Demographic Histories of White and Black Anglerfish

To investigate the demographic history of the two anglerfish species, we employed the coalescent‐based method implemented in SMC++ (Terhorst et al. 2017), which leverages whole‐genome sequencing data to infer changes in effective population size (*N*_e_) over time. SMC++ builds on the Sequentially Markovian Coalescent (SMC) framework to estimate the timing and magnitude of past population size changes by analyzing the distribution of coalescent events along the genome. For this analysis, we assumed a per‐site mutation rate of 1 × 10^−8^ mutations per generation, a commonly used estimate for teleost fish (Lynch 2010). The whole‐genome data for 14 black anglerfish (from Algarve) and for 14 white anglerfish (from Landegode) were used for demographic history reconstruction for the respective species.

## 3 Results

### 3.1 Detection of Black Anglerfish and White × Black Hybrids

The filtered data comprised 231 SNPs in 897 individuals with a genotype completeness of 92.3%. Proportions of missing genotypes were typically low to moderate (< 10%) except for a few samples (cf. Figure S2), probably because of lower DNA quality.

**FIGURE 2:**
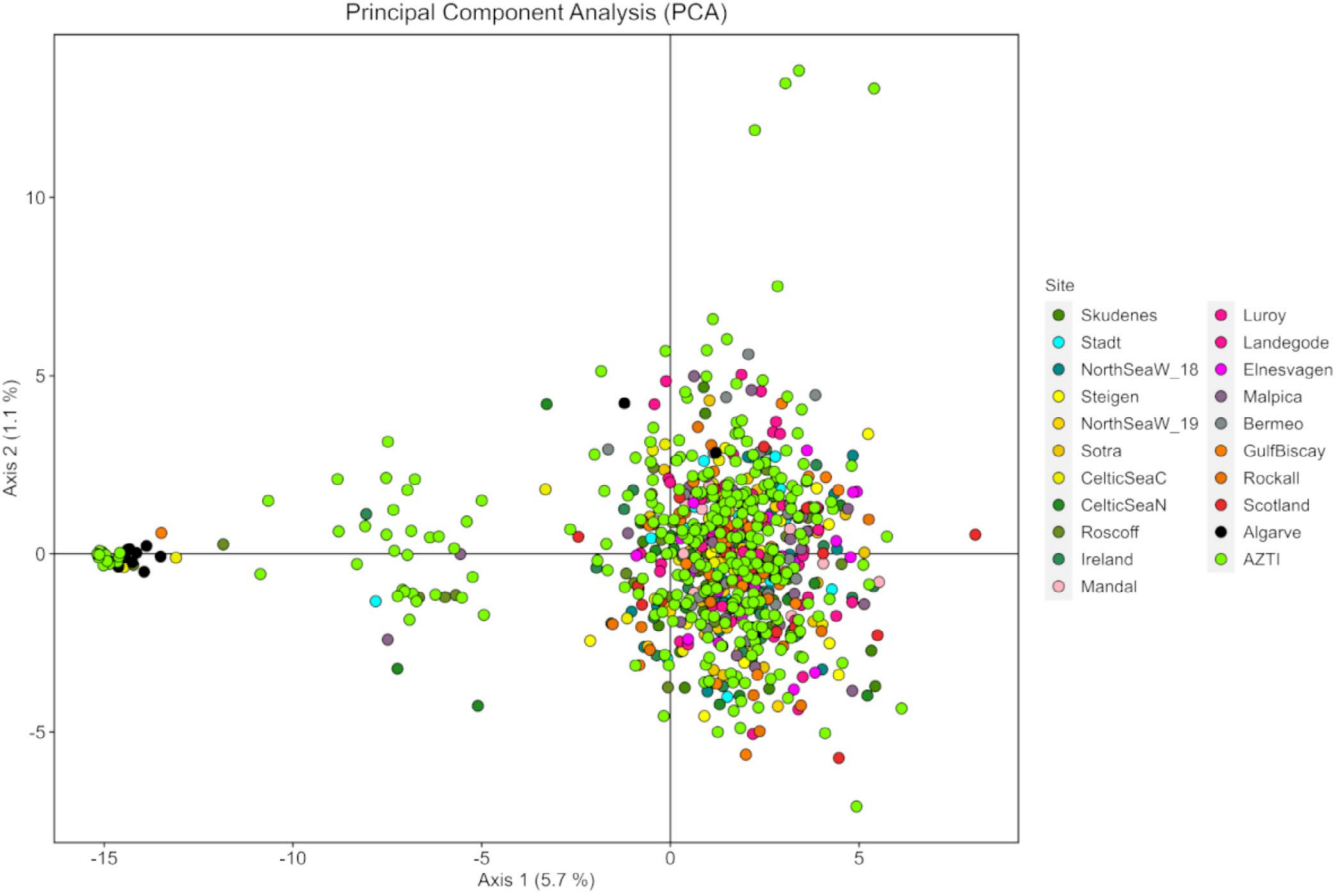
Results from the principal component analysis of 897 anglerfish genotyped at 231 SNPs. Two distinct clusters are observed, corresponding to white and black anglerfish (right and left, respectively). Species assignment is based on co‐clustering with reference individuals from Aguirre‐Sarabia et al. (2021) (light green dots), whose taxonomic identity was independently validated using mitochondrial cytochrome‐b markers. Individuals positioned intermediately between the two clusters are interpreted as putative hybrids (white × black anglerfish), consistent with admixed genomic ancestry.

**FIGURE 3:**
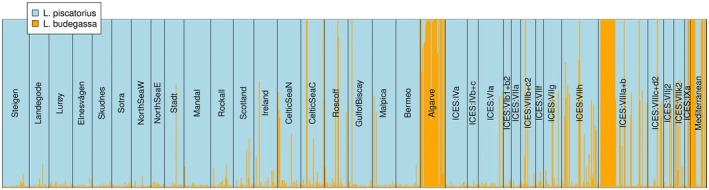
Results of the STRUCTURE analysis (ADMIX model, K=2) showing genetic clustering of black (orange bars) and white anglerfish (light blue). Individuals putatively identified as hybrids displayed intermediate bars of both colors, indicating mixed or uncertain ancestry.

PCA plots based on whole‐genome data for four sample localities (marked with asterisks in Table 1 and plotted with triangle symbols in Figure 1) revealed two distinct clusters, one consisting of individuals from Algarve, the other of individuals from Santander, Mandal, and Landegode (Figure S3). On the other hand, the much larger 231 SNP dataset revealed three clusters (Figure 2), a pattern that is compatible with the presence of both white and black anglerfish and their hybrids in the dataset, as previously identified by Aguirre‐Sarabia et al. (2021). STRUCTURE at *K* = 2 (Figure 3) allowed the separation of the data into species for downstream analyses: Using a cut‐off *q*‐value of 0.93 yielded 70 black anglerfish, 772 white anglerfish, and left 55 individuals unclassified. As these 55 individuals displayed intermediate *q*‐values between both species, a putative interpretation would be that they represent interspecific white × black anglerfish hybrids, in line with earlier findings (Aguirre‐Sarabia et al. 2021). The geographic distributions of the two pure species and putative hybrids (Figure S4) revealed particularly high occurrences of black anglerfish in the Mediterranean, in the SW of the Iberian Peninsula, in the Bay of Biscay and north to the Celtic Sea. Most hybrids occurred from Bay of Biscay to northwestern UK, with only a few individuals elsewhere and none in the North Sea basin. A single putative hybrid was found along the Norwegian coast, at Stadt.

A closer inspection of the distribution of individual *q*‐values (Figure S5) revealed that intermediate *q*‐values tended to concentrate within a rather narrow peak centered on the intermediate *q* = 0.5 value (range: ca 0.4–0.6). This peak represents the expected value for first generation (F1) hybrids and indicates that these individuals are white × black anglerfish hybrids. A few individuals fall outside this central peak, having *q*‐values in the ranges 0.1–0.4 and 0.6–0.9 (approx.) and might indicate backcrosses with parental black and white anglerfish, respectively. However, these individuals tended to have higher than average percentages of missing genotypes (most easily seen in Figure S5c), thus making their classification uncertain.

### 3.2 White Anglerfish Population Structure

The removal of black anglerfish and putative hybrids (or uncertain) individuals left 772 white anglerfish individuals for analysis of population structure. Grouped into 35 collections for statistical analysis, expected heterozygosity, averaged over the 231 SNP genotypes, was similar among samples over the entire study area and ranged from *H*_S_ = 0.297–0.341 (Table 1, right‐hand side) with *H*_T_ = 0.328 in the pooled (*n* = 772) sample. *F*_IS_ estimates ranged from −0.062 to 0.069 with *F*_IT_ = 0.005 in the pooled sample. Pairwise *F*_ST_ estimates among samples based on whole‐genome data were 0.015 (Table S1). For the 231 SNP genotypes, *F*_ST_ (Table S2) ranged from −0.0087 to 0.0932. Several patterns in the (unadjusted) test results were evident: Most strikingly, the Mediterranean sample displayed an average pairwise *F*_ST_ to the other localities of 0.0690 and was significantly different from all of them, also after FDR adjustment for multiple testing. Although no other sample pairs retained significant results after FDR adjustment, the ICES area IXa (located off Portugal) differed from the majority of other samples, with an average pairwise *F*_ST_ of 0.0139, and was significantly different in 24 out of 34 pairwise comparisons before FDR adjustment. Also, Elnesvagen, Skudenes, the eastern North Sea, Rockall, Scotland, and some of the ICES areas tentatively showed genetic differentiation from several other locations.

No signal of isolation by distance (IBD) was detected in our data (Figure S6), regardless of any depth constraints imposed on movement trajectories and thereby distances (cf. Figure S6). However, grouping samples into ICES assessment areas revealed statistically significant allele frequency differences between the Central and Northern Shelf units, although at a very low level (*F*_ST_ = 0.0005; Table 2). A similarly low‐level differentiation was also observed for the Southern versus Northern Shelf comparison but did not quite reach statistical significance (*p* = 0.0796). No pairs remained significant after FDR adjustment for multiple testing. Consistent with these findings, DAPC suggested broad overlap among these four ICES groups (Figure S7).

**TABLE 2:** Pairwise estimates of genetic difference (*F*_ST_: below diagonal) and joint tests for allele frequency difference (*p* value: above diagonal) between ICES assessment areas in the North Atlantic (*n* = sample sizes, pooling individual samples).

ICES assessment areas	Southern (*n* = 83)	Northern (*n* = 253)	Northern Shelf (*n* = 290)	Norwegian Sea (*n* = 138)
Southern	—	0.2065	0.0796	0.7796
Northern	0.0004	—	0.0089	0.3332
Northern Shelf	0.0005	0.0005	—	0.6871
Norwegian Sea	−0.0005	0.0001	−0.0003	—

Although several SNPs in the white anglerfish whole‐genome data were initially suspected as candidates for selection (Figures S8–S10), none were located within annotated functional genomic regions and were therefore not considered further as potential selected outliers herein. Arlequin revealed three candidate outliers to positive selection on white anglerfish (LOPH009, LOPH155, and LOPH248) within the set of 231 SNPs; however, BayeScan failed to confirm any of them.

### 3.3 Demographic Analyses

**FIGURE 4:**
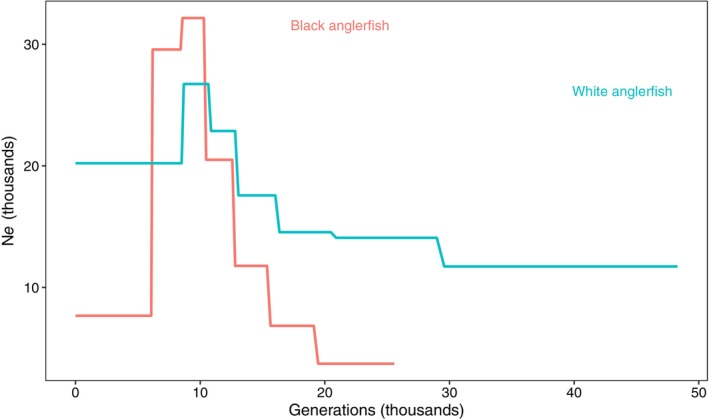
Demographic analysis of black and white anglerfish using SMC++ (Terhorst et al. 2017) based on whole‐genome data. Inferred effective population size (*N*_e_) trajectories reveal demographic changes consistent with a recent expansion in the North Atlantic (50,000–25,000 generations ago). Black anglerfish show more recent reductions and recoveries, and a lower overall *N*_e_ (~8000) compared to white anglerfish (~20,000).

Demographic analysis comparing black and white anglerfish sample collections revealed historical shifts in effective population size (*N*_e_) consistent with a relatively recent expansion of anglerfish in the North Atlantic (Figure 4), inferred between 50,000 and 25,000 generations ago. Assuming generation times of 11 and 10 years for white and black anglerfish, respectively (https://www.fishbase.se/summary/716; Froese and Pauly 2025), these estimates correspond to roughly 550,000 and 250,000 years. The inferred *N*_e_ trajectories suggest that black anglerfish populations experienced more recent reductions and recoveries than white anglerfish, dating back roughly half as far in time. Both species exhibited gradual population growth until ~10,000 generations ago, followed by an abrupt decline beginning ~6000 generations ago, down to ~8000 for black anglerfish and ~20,000 for white anglerfish, respectively.

## 4 Discussion

The current study expands on the existing genetic knowledge on white anglerfish by including whole‐genome data, increasing sampling range and sizes, and mapping SNPs to the (draft) genome of the species. We confirm previous findings of both extensive hybridization and misidentification with the closely related black anglerfish, as well as the largely homogenous genetic structure of the white anglerfish in the North Atlantic together with its divergence from the Mediterranean populations (Blanco et al. 2008; Aguirre‐Sarabia et al. 2021). Our SNP panel proved informative for resolving population structure within white anglerfish and differentiated between white and black anglerfish indicating that the SNP panel has potential as a cost‐effective tool for both species identification and population assignment in fisheries monitoring and management.

The lack, or nearly so, of spatial genetic differentiation of the North Atlantic white anglerfish is curious/intriguing, given the high occurrence of hybrids in parts of this area. Genetic uniformity challenges the expectations of hybridization leading to introgression of genes into the parent species through backcrossing, since introgression would be expected to be more pronounced in the areas where hybrids are found. A possible explanation is that we only analyzed fish that were visually identified as white anglerfish therefore obtaining an incomplete picture of the real occurrence of hybrids, which may be more geographically widespread than observed. Another explanation is that hybrids might have reduced fertility and do not interbreed successfully therefore precluding introgression. We found several individuals that seemed to be genetically intermediate between the inferred F1 hybrids and the parental species, that is, putative backcrossed individuals. However, we also found that these individuals tended to have considerably higher proportion of missing genotypes than average (cf. Figure S5), suggesting poor DNA quality and possible imprecise classification in STRUCTURE. This software (and others) is known to produce less accurate *q*‐values for individuals with missing data (Falush et al. 2003), implying that such values will tend to deviate from nominal 0, 1, and 0.5, represented by the two parental species and F1 hybrids, respectively. Thus, it is unclear if backcrosses exist, and if the two species produce fertile hybrids; in agreement with Aguirre‐Sarabia et al. (2021) who concluded that further studies are required to assess hybrid fitness.

The spatial genetic heterogeneity of white anglerfish was tested after purging the data set from black anglerfish and putative hybrids. Pairwise tests (Table S2) verified the genetic distinctness of Mediterranean and ICES area IXa (Portugal) from all or most other samples, as well as difference between these two. Curiously, the latter was the largest pairwise *F*_ST_ in the study (*F*_ST_ = 0.0932), and much larger than the average of 0.0139, but sample sizes were small and point estimates therefore uncertain. Several other pairwise estimates were also significant when judged separately, but lost significance when correcting for multiple tests using the false discovery rate (FDR) approach. These significances therefore likely represent random errors due to multiple testing and perhaps also so‐called chaotic genetic patchiness (Broquet et al. 2013; Selwyn et al. 2016) due to incomplete mixing of offspring among spawning units. Moreover, evidence for large‐scale patterns in our data was limited to weak (*F*_ST_ = 0.0005) but significant differences between the pooled samples from the areas currently defined by ICES as the Central and Northern Shelf stocks. Because of multiple testing this test result may be spurious, however, and should be regarded as tentative until confirmed with additional data. Consequently, we found no evidence that geographic distance, whether calculated as shortest distance over water or following more elaborate routes through bathymetric restrictions (cf. Figure S6), had any notable effect on genetic differences. While such isolation by distance (IBD) patterns are commonly observed among shallow‐water or coastal marine fishes, they are often absent or weak in open‐sea species, where connectivity is shaped more by larval dispersal mechanisms and oceanographic currents rather than mere geographic proximity (for review see Baco et al. 2016).

Although spatially varying environmental factors (e.g., temperature, salinity, productivity) can generate adaptive genetic differentiation even in high gene‐flow marine fishes (Bradbury et al. 2010; Selkoe et al. 2010; Lamichhaney et al. 2012), our whole‐genome analyses found no coding‐region outliers linked to sampling locality. This could reflect limited statistical power, given few (3) sample locations and small sample sizes (13–14 per site), and thus local adaptive alleles, if present, may have been missed.

As an open‐water fish, quantitative information on spawning locations and dispersal capacity of adult and juvenile anglerfish is limited. Our very low estimates of regional divergence in the North Atlantic (*F*_ST_ 0.0005 or lower) and nearly flat regression lines on distance (Figure S6) are consistent with highly effective dispersal in white anglerfish. Computer simulations have shown that local estimates of effective populations size (*N*_e_) converge toward the global effective population when dispersal is high (> 5%–10% per generation: Waples and England 2011; Novo et al. 2023). Hence, our present estimate of *N*_e_ in white anglerfish, presently ca 20,000 (Figure 4), is likely to approach the species' total *N*_e_ in the North Atlantic, despite being calculated from fish collected from a single locality (Landegode, Norway). Convergence is not instantaneous, however: when gene flow is limited, recent generations are less representative of the global population than earlier ones, which can create the appearance of a recent decline even if the true population size remained constant (Wang and Whitlock 2003; Charlesworth 2009). This effect may contribute to the decline estimated for the most recent epoch (Figure 4), suggesting that our current *N*_e_ are not fully representative of the global effective population. Estimates of effective population size are typically much lower than census abundance, often by several orders of magnitude in marine fishes. This pattern is also evident here, as annual catches alone are on the order of 10–20 million individuals (FAO 2024). The reason(s) for this difference between effective and census numbers is a topic of disagreement and discussion: On one hand, marine fishes and other organisms are producing very large number of eggs and larvae with high average mortality, which opens for some lucky few families to leave a disproportionately large number of survivors (sweepstake reproductive success: Hedgecock and Pudovkin 2011). On the other, substantial downward bias in genetic *N*_e_‐estimates may occur due to nonrandom sampling, selection, migration, and other causes (Waples 2016). Downward bias or not, estimates of *N*_e_ for both anglerfish species give no reason for concern, as they are both well above the recommended minimum for maintaining genetic variability in the long term, typically taken to be at least 500–1000 (Frankham et al. 2014).

Mediterranean and ICES area IXa differ genetically from other white anglerfish and are therefore likely to belong to populations that may be partly or wholly isolated from conspecifics further north. More sampling is needed to characterize population structure and effective sizes in these southern parts of species' range.

The SMC++‐inferred demographic trajectories should be interpreted with caution, as estimates of historical *N*_e_ depend on assumptions about mutation rate, generation time, and model simplifications, and we did not compute bootstrap‐based confidence intervals. In addition, SFS‐based methods can be affected by reduced resolution in recent time periods (Terhorst et al. 2017; Mazet et al. 2016). Our primary aim, however, was to provide a comparative demographic perspective between the two species. Assuming that similar biases affect both datasets, relative differences in the inferred trajectories remain informative, and the overall concordance in trends supports the robustness of the comparative patterns. The inferred expansion is consistent with postglacial range expansion following the Last Glacial Maximum (Hewitt 2000), whereas the decline ~6000 generations ago may reflect mid‐Holocene environmental changes influencing habitat availability and oceanographic conditions (Albretsen et al. 2011). The more recent and pronounced fluctuations in black anglerfish may indicate greater sensitivity to environmental variability or differences in life‐history traits.

Of the 897 individuals analyzed, 125 (13.9%) were misidentified, including 70 black anglerfish mistakenly classified as white anglerfish (7.8%) and 55 hybrids (6.1%, including a few uncertain cases). This represents a significant proportion of the catch and could have direct implications for stock assessment. While we do not know if there are also white anglerfish misclassified as blacks in the fisheries, and thus perhaps modify or even reverse our observations, the observed misidentification of many black anglerfish as whites is expected to result in an underestimate of the catch of black anglerfish. Comparable patterns of misidentification have been observed in other species: rates often > 20% among closely related species in the *Sebastes* complex (Cadrin et al. 2010), and ~14% in freshwater trout and Murray cods from Australia (Lyon et al. 2018). The ~14% error rate observed here therefore falls within this documented range, underscoring the need for morphological (e.g., counting dorsal fin rays) and genetic verification protocols to improve catch reporting in mixed‐species anglerfish fisheries.

### 4.1 Implications for Stock Assessment and Management

The detection of hybrids (6.1%) introduces management complexities. First, we do not know if production of interspecific hybrids is a recurring, “natural” phenomenon or reflects recent changes in the environment or in fisheries pressure. If recent, there is the possibility that F1 hybrids, if fertile, may backcross with the parental species and lead to genetic introgressions in coming generations with unknown consequences. Second, without explicit recognition, hybrids may bias stock assessments if they are assigned to one parental species, particularly if hybridization is asymmetric and disproportionately affects the rarer black anglerfish. Several options could help mitigate this risk: (i) incorporating genetic or molecular screening tools for routine hybrid detection, (ii) recording hybrids as a separate reporting category in fisheries statistics, and (iii) investigating the impact of species misidentification and hybrids on stock assessment. Genetic differences among localities or larger regions, when they exist, generally imply that interchange (gene flow) between populations is sufficient to make them demographically independent. As pointed out by Aguirre‐Sarabia et al. (2021), white anglerfish in the Mediterranean is clearly distinct from the North Atlantic stock(s), a finding consistent with its current recognition as a separate management stock. In our study, we also find indications of genetic distinctness in Portuguese waters (ICES area IXa); however, this result remains uncertain because of small sample sizes (Algarve: *n* = 3; AZTI IXa: *n* = 5; Table 1). Among the presently recognized Atlantic stocks, the only tentative sign of genetic differences was observed between pooled samples from the Central as compared to the Northern Shelf stock. Yet, this difference is very small and is significant largely due to the large sample sizes (*n* = 253 and *n* = 290, respectively). Moreover, given the overall low level of divergence, the reality of this finding remains unclear until it can be independently verified.

Lack of evidence for genetic differences, on the other hand, might be masking a recent demographic split where populations did not have enough time to evolve detectable genetic differences using the current sampling and genotyping efforts (Waples et al. 2008). In particular, our southern (*n* = 83) and Norwegian Sea (*n* = 138) collections may be too small, or divergence too subtle, to reveal true separation. Therefore, conclusions about population structure should integrate complementary evidence such as tagging studies (e.g., Landa, Quincoces, et al. 2008), which documented limited exchange between Southern and Northern Atlantic regions; and otolith microchemistry analyses (e.g., Swan et al. 2004), which revealed geographic isolation of early‐life stages in white anglerfish. These approaches provide ecological connectivity insights on shorter timescales than genetics and can clarify population boundaries that elude the scrutiny of neutral genetic markers.

The current results supporting a panmictic or nearly panmictic population of white anglerfish throughout the Northeast Atlantic is consistent with earlier studies. However, this does not automatically mean that the entire population should be assessed in one assessment model. Gene flow sufficient to prevent genetic differentiation does not imply that the population is not spatially structured. For example, spawning patterns show clear spatial structure (ICES 2024b). Other factors determining stock productivity (growth and natural mortality) are also likely to vary regionally due to environmental factors. Finally, fishing pressure varies spatially, especially since management takes place on a much smaller spatial scale than that of the population. Most assessment models assume full spatial mixing within the assessment area (dynamic pool assumption), some models can account for spatial structure, but the added complexity of these models has prevented widespread usage so far. Detailed data on connectivity (larval and adult) between areas is necessary for a spatially structured assessment model. Without this information, the precautionary approach would suggest continuing with separate assessments by area.

Management of anglerfish is conducted jointly for both species. ICES (2024b) states that “The use of a combined species total allowable catch (TAC) for the two anglerfish species, black‐bellied anglerfish ( *Lophius budegassa* ) and white anglerfish ( *Lophius piscatorius* ), prevents effective control of the single‐species exploitation rates and could lead to the overexploitation of either species.” Implementing management for each species is challenging because catches are often reported jointly/for unseparated anglerfish. Changing reporting requirements, or at least keeping the species separate in research surveys, would be a necessary first step toward species‐specific management. Genetic tools may be necessary to accurately quantify the proportion of the two species and their hybrids in the commercial catches as well as surveys used in the assessment (e.g., Farrell et al. 2022). The implementation of these approaches on a large scale would be crucial for species‐specific management and monitoring.

## 5 Conclusions

This study establishes a genomic baseline for white anglerfish, demonstrating a largely panmictic population across the Northeast Atlantic, clear divergence from Mediterranean populations, and pervasive hybridization and misidentification with black anglerfish. The SNP panel provides a practical tool which has potential to be used for species identification and fisheries monitoring. Nonetheless, key uncertainties remain. The extent of introgression, including the frequency of backcrossing and the fertility and fitness of hybrids, is still unresolved. Moreover, subtle genetic differences between regions such as the Central and Northern Shelf require further investigation to determine whether they reflect early population structuring or demographic processes. Addressing these questions will be critical for refining stock delineation and ensuring effective, genomics‐informed fisheries management.

## Funding

This study was partially supported by the Fiskeridirektoratet. R.C. received Portuguese national funds from FCT—Foundation for Science and Technology through projects UIDB/04326/2020 (https://doi.org/10.54499/UIDB/04326/2020), UIDP/04326/2020 (https://doi.org/10.54499/UIDP/04326/2020), and LA/P/0101/2020 (https://doi.org/10.54499/LA/P/0101/2020) to CCMAR.

## Conflicts of Interest

The authors declare no conflicts of interest.

## Supporting information


**Figure S1:** Genomic positions of the 231 SNPs used in the current study.
**Figure S2:** Map of percent missing genotypes in 231 SNPs among 897 individuals from 35 localities (Table 1). Highest proportions were observed in samples Mandal, Scotland, Celtic Sea, and Gulf of Biscay with 27%–17% missing genotypes per individual on average, respectively. Most other samples had < 10% missing genotypes.
**Figure S3:** Results from the principal component analysis of 55 whole‐genome re‐sequenced anglerfish, based on 3,777,012 SNPs. The Algarve sample (a subsample of *n* = 15 from this locality) clearly separates from the rest and inferred to represent black anglerfish ( *L. budegassa* ).
**Figure S4:** Geographic distribution of pure species and putative hybrids identified by STRUCTURE genetic clustering of 231 SNPs. Pie charts, depicting proportion of the three types, are plotted at the average geographic positions of individuals belonging to each of 35 geographic collections (cf. Table 1) and scaled in size to number of individuals.
**Figure S5:** Comparison of individual STRUCTURE *q*‐values (admix model) with information on percentages of missing genotypes per individual. (a) Number of individuals per *q*‐value bin. (b) Same as (a) with different vertical scale for clarity. (c) Relative proportion of individuals per *q*‐value bin. Note that individuals with intermediate *q*‐values in some ranges (ca 0.1–0.4 and 0.6–0.9) tended to have large percentages of missing genotypes (colored) and may be considered uncertain.
**Figure S6:** Analyses of isolation by distance (IBD) using pairwise *F*_ST_ (Table S2) among *L. piscatorius* samples against distance over water: (a, b) Depth constrained to 20–1000 m (appropriate for adult movements, although some individuals have been observed crossing very deep areas); (c, d) No restriction on depth (e.g., larval drift). No significant trends were observed (Mantel test *p* values, 0.13 and 0.20, respectively). The Mediterranean sample was excluded from both analyses because genetic results indicated it represents a distinct group.
**Figure S7:** Result from the DAPC analysis. A low differentiation among four ICES groups was suggested. Sample sizes for the Northern, Northern shelf, Norwegian Sea, and Southern clusters were 253, 290, 138, and 83 (also see Table 2).
**Figure S8:** Pairwise *F*_ST_ values between Landegode and Mandal samples estimated from whole‐genome data across 23 chromosomes. The horizontal black dashed line shows the genome‐wide mean *F*_ST_ = 0.016, the red dots represent SNPs with estimates of *F*_ST_ exceeding the 99.95th percentile.
**Figure S9:** Pairwise *F*_ST_ values between Landegode and Santander estimated from whole‐genome data across 23 chromosomes. The horizontal black dashed line shows the genome‐wide mean *F*_ST_ = 0.015, the red estimates are the *F*_ST_ values exceeding the 99.95th percentile.
**Figure S10:** Pairwise *F*_ST_ values between Landegode and Mandal estimated from whole‐genome data across 23 chromosomes. The horizontal black dashed line shows the genome‐wide mean *F*_ST_ = 0.016, the red estimates are the *F*_ST_ values exceeding the 99.95th percentile.
**Table S1:** Pairwise *F*_ST_ (Weir and Cockerham 1984) estimates among the four studied samples based on total 3,777,012 SNPs derived from whole‐genome data. Sample sizes are shown in brackets. Note that some, but not all, individuals included in the whole‐genome sequencing dataset from Algarve (*n* = 14), Santander (*n* = 8), Mandal (*n* = 9), and Landegode (*n* = 8) were also included in the SNP genotyping dataset.
**Table S2:** Pairwise *F*_ST_ (Weir and Cockerham 1984; above the diagonal) estimates among the 35 sample collections based on total 231 SNPs. Values in red are statistically significant (*p* < 0.05; *p* values presented below the diagonal) as per the allele frequency tests. Black anglerfish and tentative hybrids were excluded from this analysis. The sample collection from ICES:VIIId2 was excluded as most of these fish appeared as black anglerfish.

## Data Availability

The raw whole‐genome sequencing data are publicly available through NCBI under submission number SUB16142129. Additional biological and SNP array data are accessible via the Dryad repository: https://doi.org/10.5061/dryad.kd51c5bnc.
