# Supplementary material for: Genomic Insights Into Population Structure and Demographic History of White Anglerfish ( Lophius piscatorius ) Throughout Its Range
Source: Evol Appl. 2026 Jul 25;19(7):e70288. doi: 10.1111/eva.70288 (PMC13401689; doi:10.1111/eva.70288)
Supplement: Supplementary file 1 — Figure S1: Genomic positions of the 231 SNPs used in the current study. Figure S2: Map of percent missing genotypes in 231 SNPs among 897 individuals from 35 localities (Table 1). Highest proportions were observed in samples Mandal, Scotland, Celtic Sea, and Gulf of Biscay with 27%–17% missing genotypes per individual on average, respectively. Most other samples had < 10% missing genotypes. Figure S3: Results from the principal component analysis of 55 whole‐genome re‐sequenced anglerfish, based on 3,777,012 SNPs. The Algarve sample (a subsample of n = 15 from this locality) clearly separates from the rest and inferred to represent black anglerfish ( L. budegassa ). Figure S4: Geographic distribution of pure species and putative hybrids identified by STRUCTURE genetic clustering of 231 SNPs. Pie charts, depicting proportion of the three types, are plotted at the average geographic positions of individuals belonging to each of 35 geographic collections (cf. Table 1) and scaled in size to number of individuals. Figure S5: Comparison of individual STRUCTURE q‐values (admix model) with information on percentages of missing genotypes per individual. (a) Number of individuals per q‐value bin. (b) Same as (a) with different vertical scale for clarity. (c) Relative proportion of individuals per q‐value bin. Note that individuals with intermediate q‐values in some ranges (ca 0.1–0.4 and 0.6–0.9) tended to have large percentages of missing genotypes (colored) and may be considered uncertain. Figure S6: Analyses of isolation by distance (IBD) using pairwise F ST (Table S2) among L. piscatorius samples against distance over water: (a, b) Depth constrained to 20–1000 m (appropriate for adult movements, although some individuals have been observed crossing very deep areas); (c, d) No restriction on depth (e.g., larval drift). No significant trends were observed (Mantel test p values, 0.13 and 0.20, respectively). The Mediterranean sample was excluded from both analyses because ge [file EVA-19-e70288-s001.docx]

**Supplementary Information**

**Genomic insights into population structure and demographic history of white anglerfish (Lophius piscatorius) throughout its range**

**TABLES**

**Table S1** Pairwise *F*_ST_ (Weir & Cockerham 1984) estimates among the four studied samples based on total 3 777 012 SNPs derived from whole-genome data. Sample sizes are shown in brackets. Note that some, but not all, individuals included in the whole-genome sequencing dataset from Algarve (n = 14), Santander (n = 8), Mandal (n = 9), and Landegode (n = 8) were also included in the SNP genotyping dataset.

|  | **Algarve (14)** | **Santander (14)** | **Mandal (13)** |
| --- | --- | --- | --- |
| **Santander (14)** | 0.168 |  |  |
| **Mandal (13)** | 0.169 | 0.015 |  |
| **Landegode (14)** | 0.168 | 0.015 | 0.015 |

**Table S2** Pairwise *F*_ST_ (Weir & Cockerham 1984; above the diagonal) estimates among the 35 sample collections based on total 231 SNPs. Values in red are statistically significant (*p*<0.05; *p* values presented below the diagonal) as per the allele frequency tests. Black anglerfish and tentative hybrids were excluded from this analysis. The sample collection from ICES:VIIId2 was excluded as most of these fish appeared as black anglerfish.

**FIGURES**

**
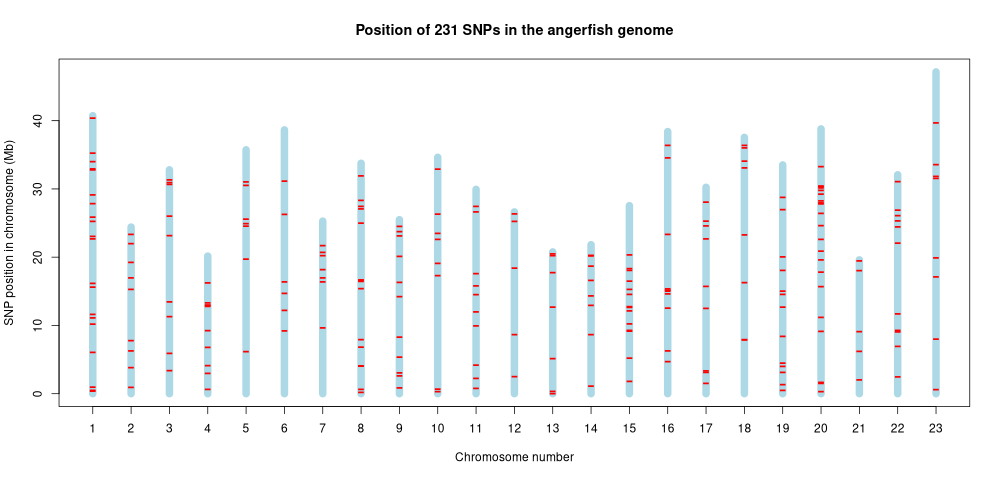
**

**Figure S1** Genomic positions of the 231 SNPs used in the current study.


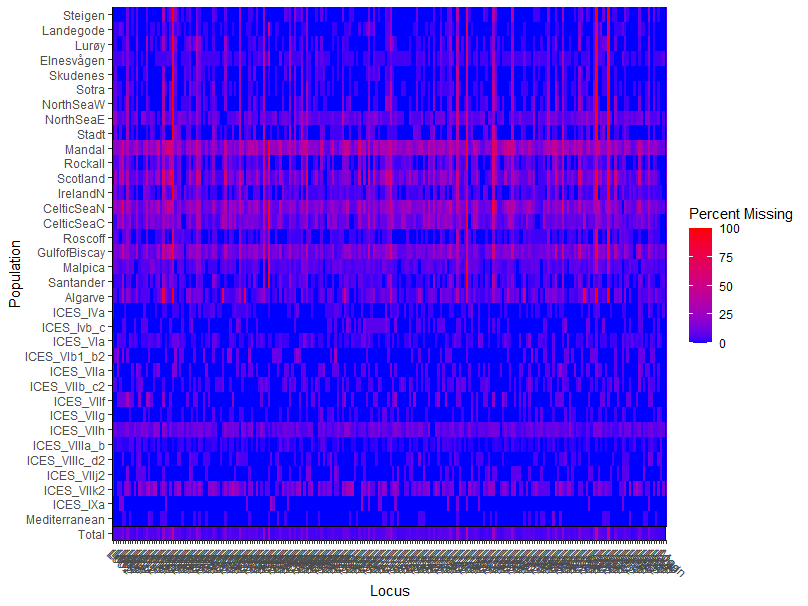


**Figure S2** Map of percent missing genotypes in 231 SNPs among 897 individuals from 35 localities (Table 1). Highest proportions were observed in samples Mandal, Scotland, Celtic Sea and Gulf of Biscay with 27% to 17% missing genotypes per individual on average, respectively. Most other samples had <10% missing genotypes.

**Figure S3** Results from the principal component analysis of 55 whole-genome re-sequenced anglerfish, based on 3 777 012 SNPs. The Algarve sample (a subsample of n=15 from this locality) clearly separates from the rest and inferred to represent black anglerfish (*L. budegassa*).


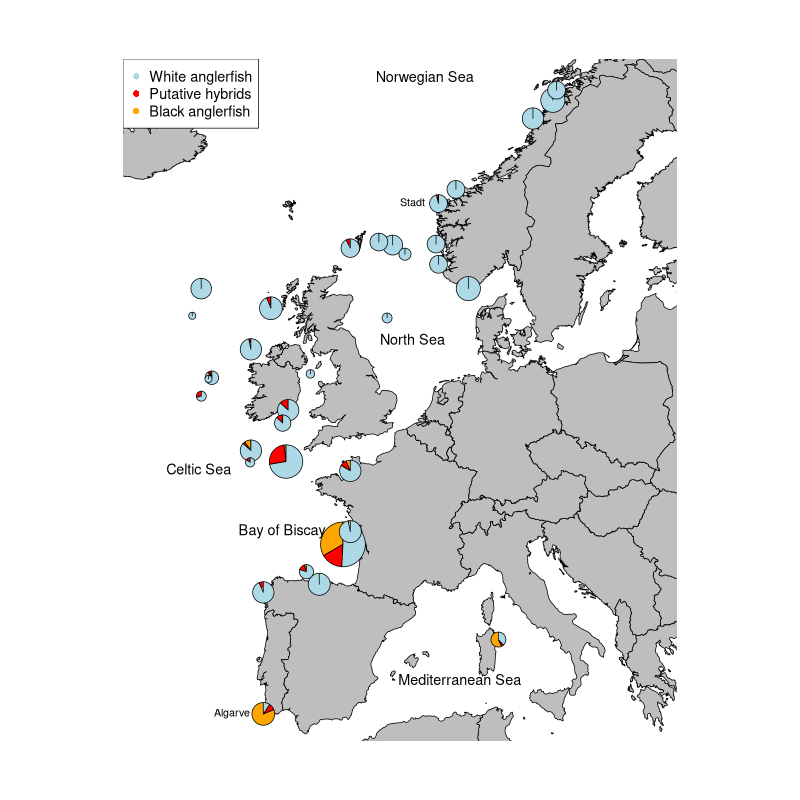


**Figure S4** Geographic distribution of pure species and putative hybrids identified by STRUCTURE genetic clustering of 231 SNPs. Pie charts, depicting proportion of the three types, are plotted at the average geographic positions of individuals belonging to each of 35 geographic collections (cf. Table 1) and scaled in size to number of individuals.


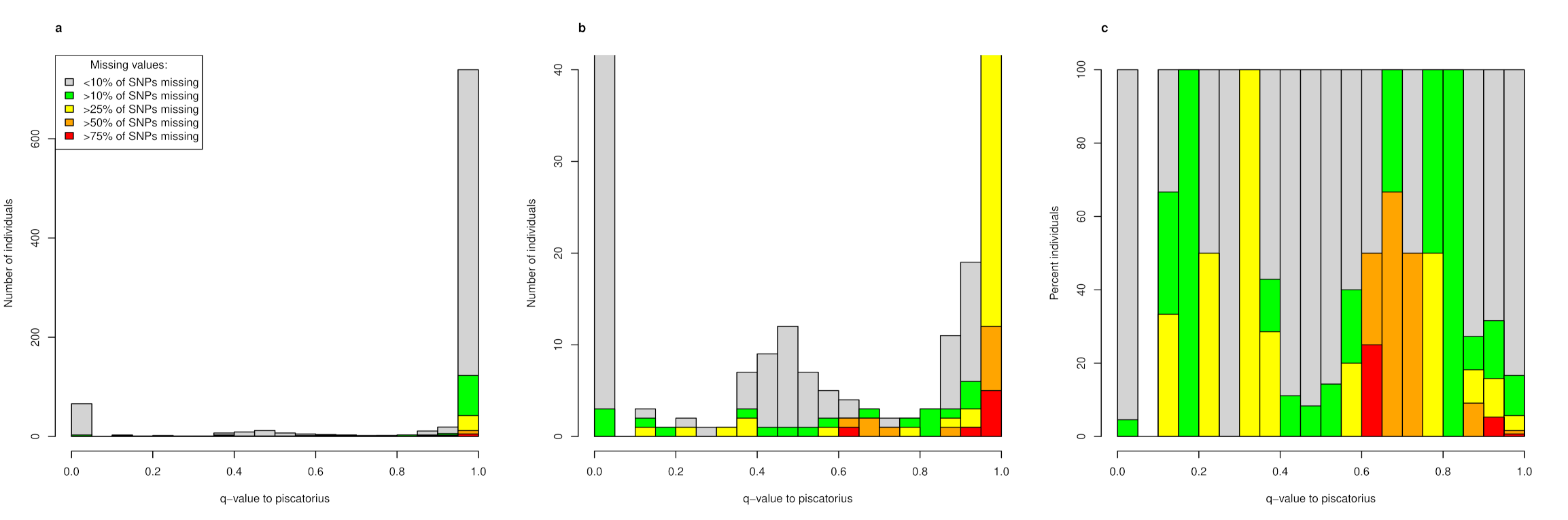
​​

**Figure S5** Comparison of individual STRUCTURE q-values (admix model) with information on percentages of missing genotypes per individual. **a)** Number of individuals per q-value bin. **b)** Same as (a) with different vertical scale for clarity. **c)** Relative proportion of individuals per q-value bin. Note that individuals with intermediate q-values in some ranges (ca 0.1 to 0.4, and 0.6 to 0.9) tended to have large percentages of missing genotypes (colored) and may be considered uncertain.


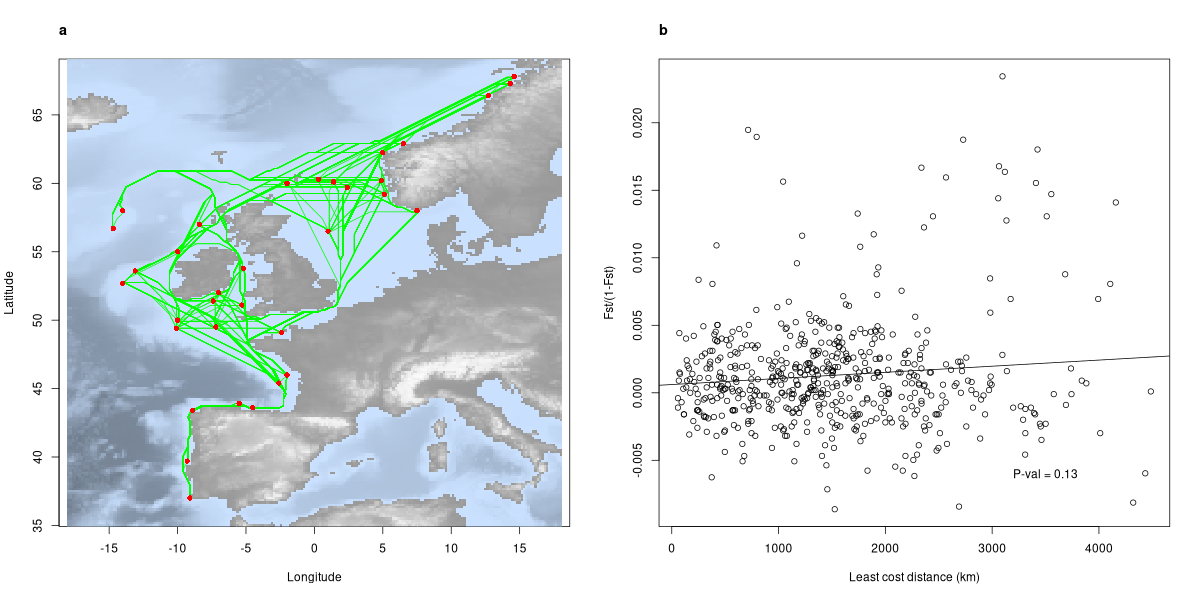


c

d


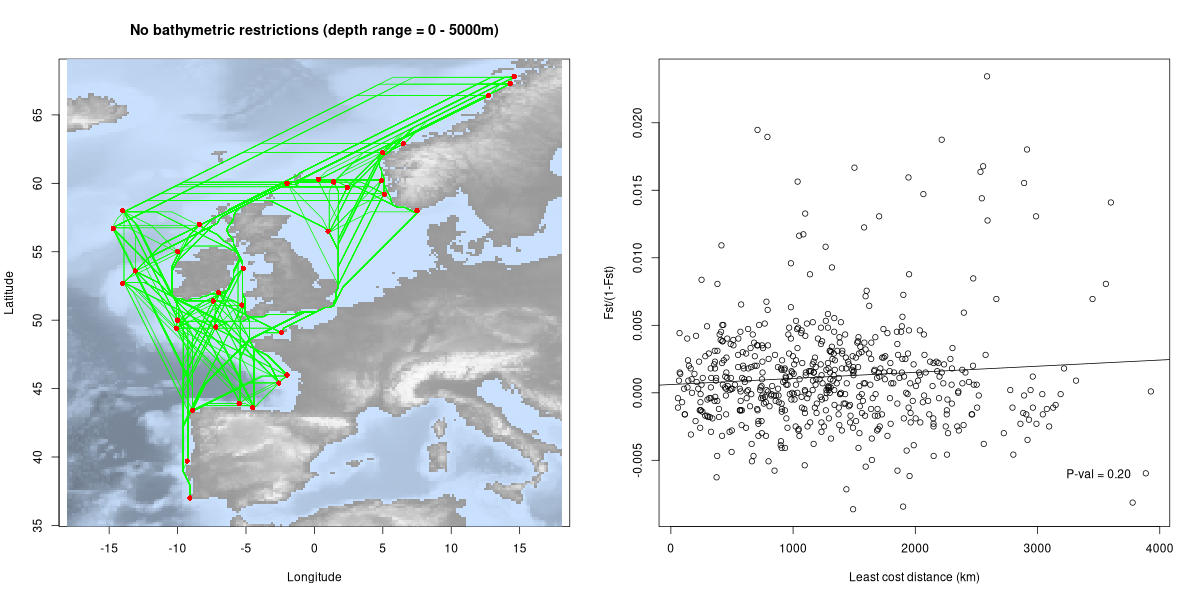


**Figure S6** Analyses of isolation by distance (IBD) using pairwise *F*_ST_ (Suppl. Table S2) among *L. piscatorius* samples against distance over water: **a, b)** Depth constrained to 20–1000 m (appropriate for adult movements, although some individuals have been observed crossing very deep areas); **c, d)** No restriction on depth (e.g. larval drift). No significant trends were obsereved(Mantel test P-values, 0.13 and 0.20, respectively). The Mediterranean sample was excluded from both analyses because genetic results indicated it represents a distinct group.


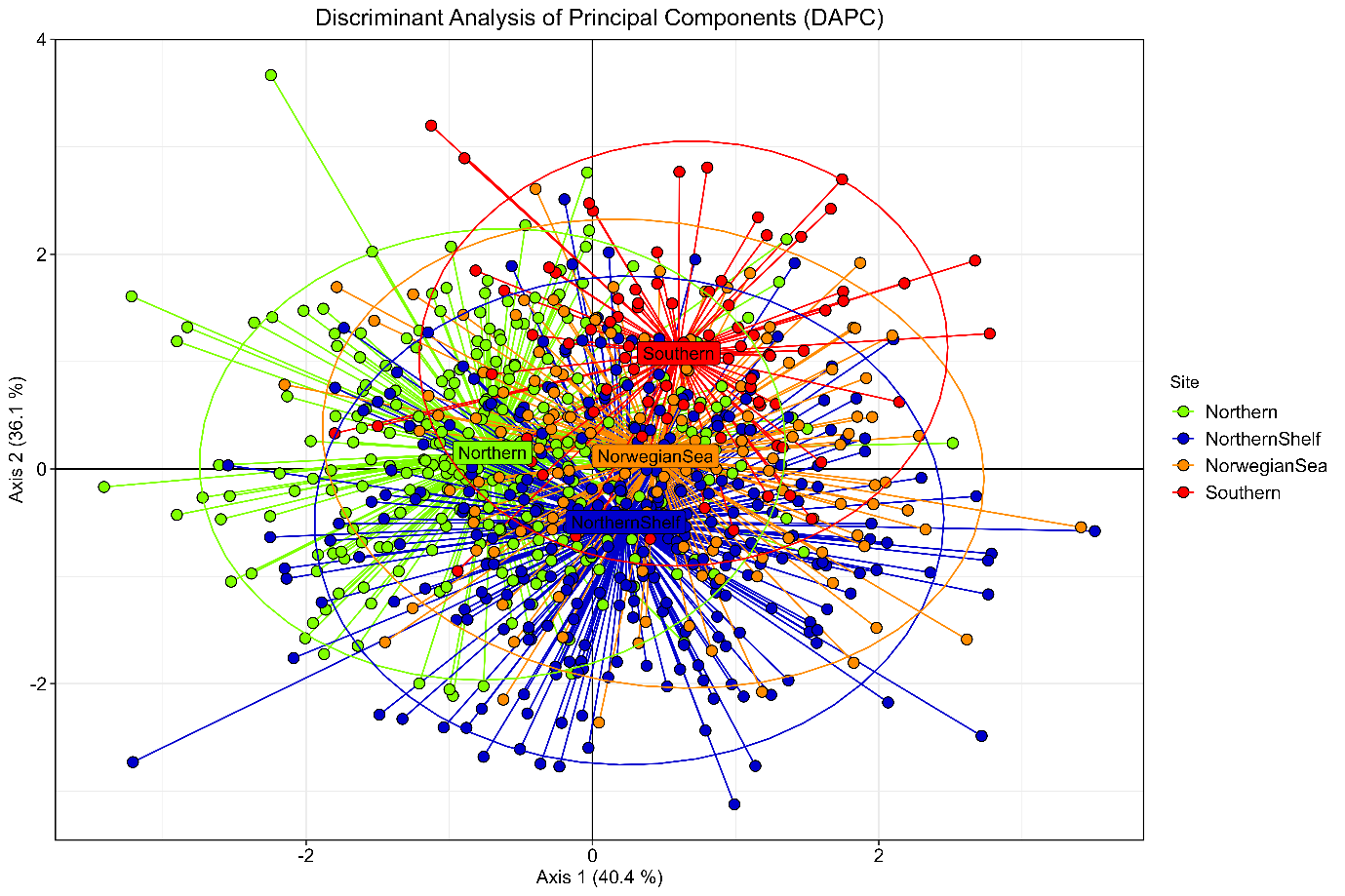
**Figure S7** Result from the DAPC analysis. A low differentiation among four ICES groups was suggested. Sample sizes for the Northern, Northern shelf, Norwegian Sea, and Southern clusters were 253, 290, 138, and 83 (also see Table 2).


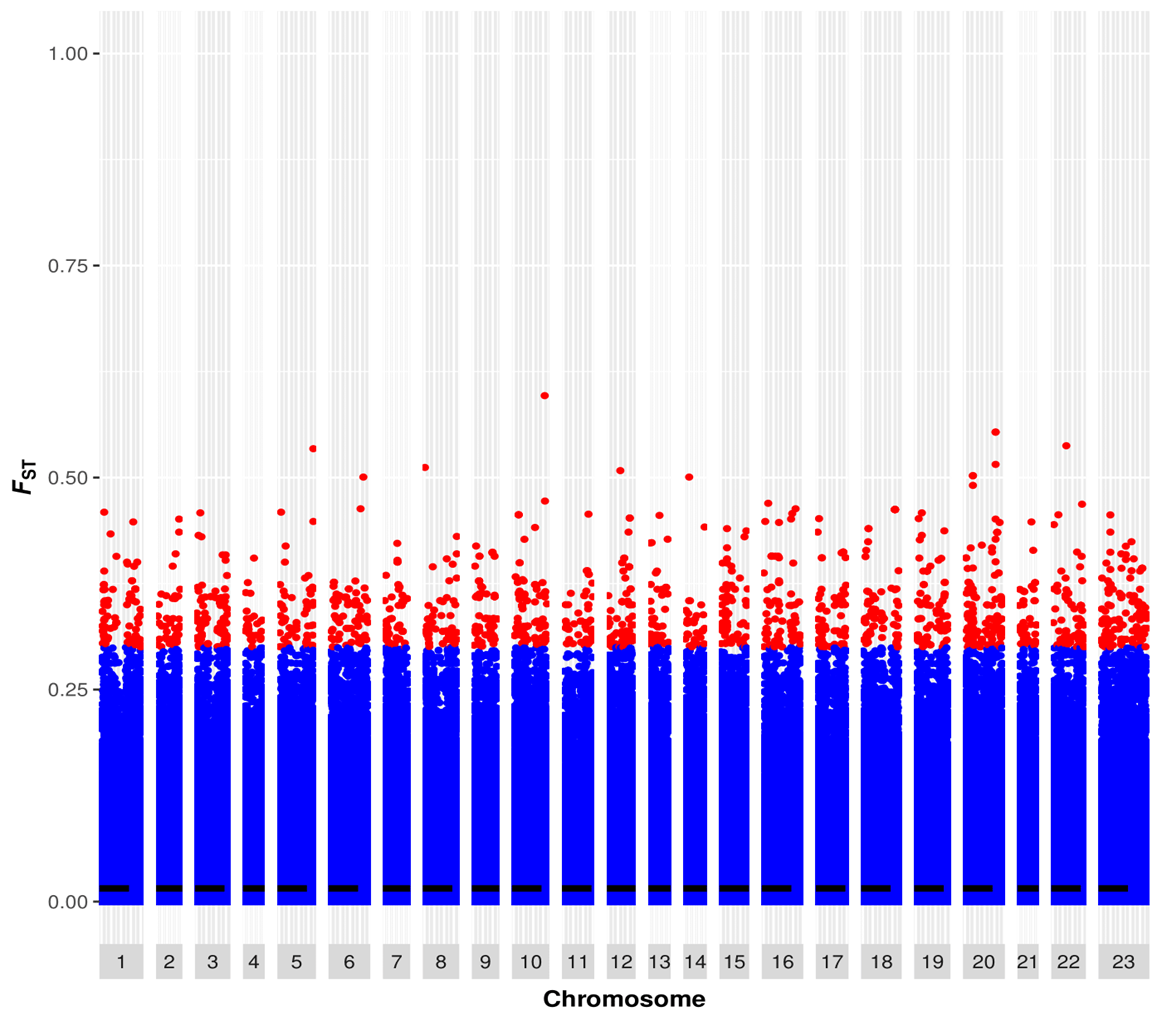


**Figure S8** Pairwise *F*_ST_ values between Landegode and Mandal samples estimated from whole-genome data across 23 chromosomes. The horizontal black dashed line shows the genome-wide mean *F*_ST_=0.016, the red dots represent SNPs with estimates of *F*_ST_ exceeding the 99.95^th^ percentile.


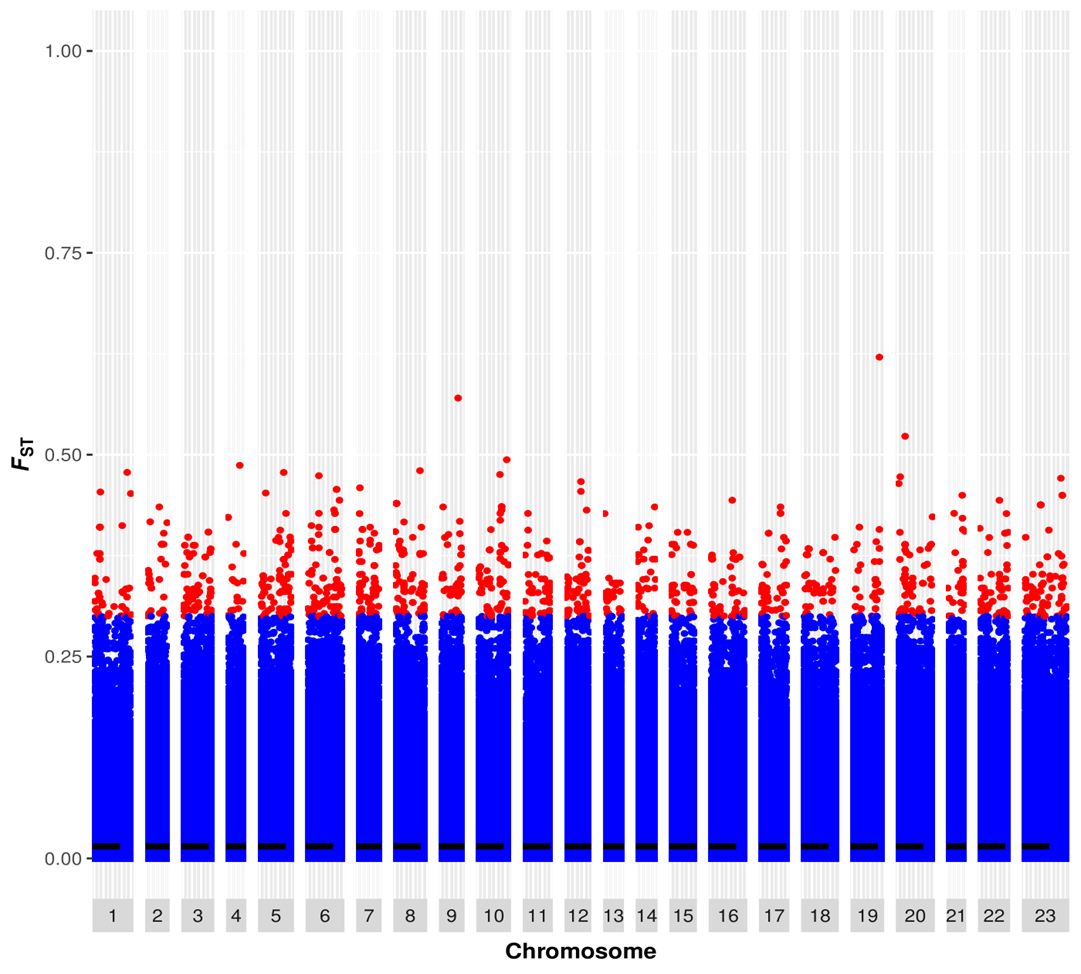


**Figure S9** Pairwise *F*_ST_ values between Landegode and Santander estimated from whole-genome data across 23 chromosomes. The horizontal black dashed line shows the genome-wide mean *F*_ST_=0.015, the red estimates are the *F*_ST_ values exceeding the 99.95^th^ percentile.


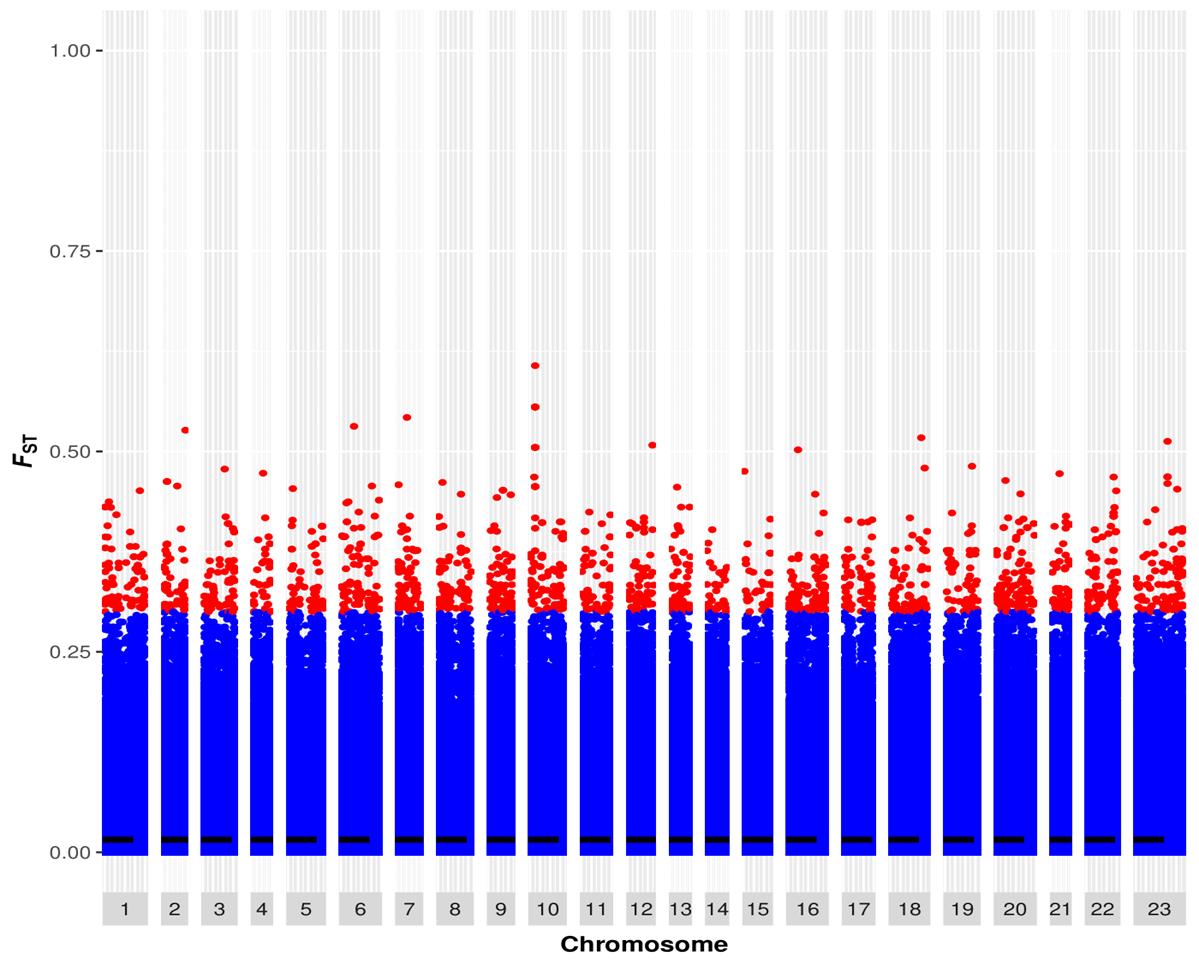


**Figure S10** Pairwise *F*_ST_ values between Landegode and Mandal estimated from whole-genome data across 23 chromosomes. The horizontal black dashed line shows the genome-wide mean *F*_ST_=0.016, the red estimates are the *F*_ST_ values exceeding the 99.95^th^ percentile.
